# *In vitro* disappearance characteristics of selected categories of commercially available dog treats*

**DOI:** 10.1017/jns.2014.40

**Published:** 2014-10-10

**Authors:** Maria R. C. de Godoy, Ryan Vermillion, Laura L. Bauer, Ryan Yamka, Nolan Frantz, Tim Jia, George C. Fahey, Kelly S. Swanson

**Affiliations:** 1 Department of Animal Sciences, University of Illinois, Urbana, IL 61801, USA; 2 Division of Nutritional Sciences, University of Illinois, Urbana, IL 61801, USA; 3 The Hartz Mountain Corporation, Secaucus, NJ 07094, USA

**Keywords:** *In vitro* disappearance, Treats, Canine nutrition, Safety, DMD, DM disappearance

## Abstract

Pet owners desire treats with adequate nutritional profiles, functional benefits, long-lasting properties and an interactive nature. Therefore, it is pivotal to understand the digestion characteristics of treats produced by different processing methods and having variable nutritional composition. The objective of the present study was to measure *in vitro* disappearance characteristics of selected categories of commercially available treats. *In vitro* procedures developed by Boisen and Eggum in 1991 were modified to handle larger sample sizes. Treat samples were evaluated in triplicate. Following incubation, *in vitro* DM disappearance (DMD) was calculated. *In vitro* DMD of selected treats varied widely. For the gastric phase, DMD ranged from 6·9 to 88·8 %, whereas intestinal phase digestion resulted in a DMD range of 10·7–100·0 % (*P* < 0·05). Because of differences in treat composition and size, they were divided into six categories: Biscuit, Bone, Chew, Dental, Meat Product and Rawhide. In general, Bone was the least digestible treat category in both gastric and intestinal phases. Meat Product and Rawhide treats had a DMD of 71·5–100 % after the intestinal phase, whereas Biscuit had values above 93 %. Chew and Dental treats had a wide DMD range (54·5–100 %). Understanding the DMD of commercially available treats is important to verify their safety for consumption and potential digestibility once ingested. These data indicate wide variation in DMD among and within different treat categories. This information will assist pet food sale associates, pet owners and veterinarians to make more educated decisions when it relates to selection and recommendations about commercially available treats. Future work is needed to expand the knowledge on *in vitro* DMD and safety of treats and to further investigate their impact on *in vivo* DM digestibility once fed to dogs.

The pet treat industry has 3 billion dollars in retail sales annually, representing a significant share (about 15 %) of the pet food market. Since 2010, an average increase of approximately 11 % has been observed in the sale of dog and cat treats^(^^1^^)^. Pet owners have high expectations for the types and attributes of pet treats, which makes the pet treat market a very dynamic and innovative sector. Treats often are used as a means to reinforce an emotional bond or to interact with the pet, as well as to deliver functional or health benefits. Meat treats are the most popular treat category, whereas the natural-flavour knotted rawhide is the most common chew treat purchased by pet owners^(^^2^^)^.

The increase in the consumption and popularity of treats raises awareness about the safety of these products upon ingestion and also about their potential nutritional and energetic contribution to the pet's diet. Safe treats must be partially or completely digested in the gastric and intestinal phases to avoid gastrointestinal blockage. They also must not result in sharp edges after chewing in order to prevent oral and gastrointestinal perforations. An effective method to test for these characteristics without risking the animal's wellbeing and health is through an *in vitro* assay technique. The test mimics the pet's digestive processes by simulating the gastric and small intestinal conditions. Therefore, the objective of the present study was to measure *in vitro* DM disappearance (DMD) characteristics of selected categories of commercially available treats.

## Materials and methods

### Substrates

A total of nineteen commercially available dog treats (The Hartz Mountain Corporation) were tested in this *in vitro* study. Treats were classified into six categories based on their appearance, size and functionality. The treat categories were: Biscuit, Bone, Chew, Dental, Meat Product and Rawhide. The Biscuit category was comprised three treats: rawhide with sweet potato, rawhide with carrot and sweet potato and co-extruded biscuit. In the Bone category, cooked pork humerus and beef femur were tested. Edible bone made with no gluten, semi-moist strips and semi-moist rolls comprised the Chew category, whereas the Dental category consisted of edible pork bone with no wheat gluten, edible bone with wheat gluten and co-extruded bone. Meat Products were described as whole pig ear, extruded rawhide braid, soft chicken jerky, chicken jerky and duck jerky; whereas the Rawhide category was comprised pig skin twist, knotted rawhide bone and extruded rawhide bone (Supplementary Table 1 – online).

### Multiple enzymatic filtration system *in vitro* method

*In vitro* DMD was determined based on the method developed by Boisen and Eggum^(^^3^^)^, but it was modified to handle large sample sizes. Briefly, 250 ml of phosphate buffer (0·1 M) and 100 ml of HCl–pepsin solution were added to each flask containing one treat to simulate gastric digestion. In addition, 5 ml of chloramphenicol was added to each flask, which was then sealed with a rubber stopper and incubated at 39°C for 6 h. Following the gastric phase, 0·5 m of NaOH was used to neutralise the HCl–pepsin solution and 100 ml of pancreatin: phosphate buffer (pH 6·8, 0·2 M, 39°C) was added; this step mimics the enzymatic digestion in the small intestine. After 18 h of incubation at 39°C, the contents of each flask were filtered through polyester fabric. Recovered residues were dried at 57°C to determine DMD.

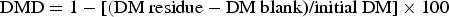

In addition to DMD determination, photographs were taken to provide pictorial representation of treat disintegration (Supplementary Fig. 1 – online).

### Statistical analysis

Data were analysed using the MIXED procedure of Statistical Analysis Systems 9·3 (SAS Institute Inc., Cary, NC). The statistical model included the fixed effect of treat type, category and *in vitro* phase. Treatment least-squares means were compared with each other and a Tukey adjustment was used to control the experimentwise error. A probability of *P* ≤ 0·05 was considered statistically significant.

## Results and discussion

**Fig. 1.**
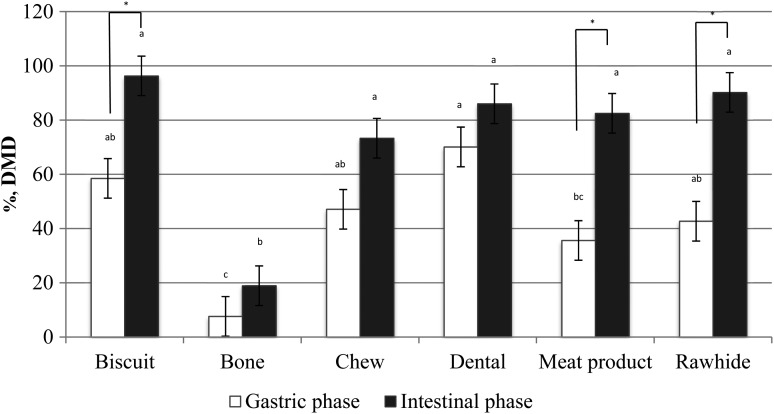
Average *in vitro* DMD by category of commercially available dog treats. *Average mean treat category effect between *in vitro* phases (*P* < 0·05). ^a–c^Average mean values for treat categories within *in vitro* phase not sharing common superscript letters differ (*P* < 0·05).

*In vitro* DMD of selected treats varied widely. For the gastric phase, DMD ranged from 6·9 to 88·8 %, whereas intestinal phase digestion resulted in a DMD range of 10·7–100·0 % (*P* < 0·05; Table 1). In the Biscuit category, similar DMD values were observed among treats in the gastric and intestinal phases. Treats in this category had a high gastric and intestinal digestibility varying from 51·5 to 68·9 % and 93·9 to 98·8 %, respectively. At the gastric phase, the Biscuit treats were partially disintegrated, which indicates that these treats would not offer risk of gastrointestinal blockage upon consumption by pets. Bones were the least digestible treat category in both gastric and intestinal phases (Fig. 1), with no improvement in DMD observed over time (Table 1). No sharp edges were observed after *in vitro* incubation of Bone treats. Even though smaller fragmented pieces were observed, they were round shaped and a result of disintegration of the softer areas of the bone (e.g. cartilage, flesh and bone marrow). Collectively, these outcomes suggest that these treats would likely not perforate the gastrointestinal tract of pets upon their consumption, but could lead to blockage if very large chunks of bone were swallowed.

**Table 1.** *In vitro* gastric and intestinal DM disappearance (DMD) of dog treats

Treat categories	Gastric phase 6 h	Intestinal phase 18 h
Biscuit		
Biscuit 1	68·9^b^	98·8^a^
Biscuit 2	55·0^b^	96·2^a^
Biscuit 3	51·5^b^	93·9^a^
Bone		
Bone 1	8·4	27·2
Bone 2	6·9	10·7
Chew		
Chew 1	85·0^A^	100·0^A^
Chew 2	27·9^bB^	65·5^aB^
Chew 3	28·3^bB^	54·5^aB^
Dental		
Dental 1	88·4^A^	95·9^A^
Dental 2	33·3^bB^	62·2^aB^
Dental 3	88·8^A^	100·0^A^
Meat Product		
Meat 1	14·3^bB^	90·1^aAB^
Meat 2	42·3^bA^	99·8^aA^
Meat 3	42·7^bA^	73·3^aB^
Meat 4	37·3^bA^	78·0^aB^
Meat 5	41·3^bA^	71·5^aB^
Rawhide		
Rawhide 1	14·2^bC^	77·5^aB^
Rawhide 2	73·1^bA^	99·5^aA^
Rawhide 3	40·9^bB^	98·7^aA^

^a–b^Means without a common superscript letter within a row differ (*P* < 0·05).

^A–C^Means without a common superscript letter within a column and treat category differ (*P* < 0·05).

sem = 3·5562.

Chew treats were highly variable in DMD in both gastric and intestinal phases, varying from 27·9 to 85·0 % and 54·5 to 100·0 %, respectively (Table 1). Chew 1 was completely disintegrated after 6 h of *in vitro* incubation, whereas Chew 2 and 3 were only partially digested. After 18 h of *in vitro* incubation, Chew 3 maintained most of its shape. Because of its soft texture and small size, however, it is not likely to pose any safety issues for pets. Dental treats were, in general, highly digestible at gastric and intestinal phases (>88 %), except for Dental treat 2 that had a DMD of 33·3 and 62·2 %, respectively. The data from the Chew and Dental categories suggest that the absence of gluten in the manufacturing of treats facilitates their DMD, which in the animal might translate to a higher digestibility and lower likelihood of gastrointestinal blockage or distress. It is important to consider that the variation in treat recipes may result in different interactions among ingredients and in the final product digestibility. Therefore, the use of wheat gluten in treat recipes may not always translate in lower DMD, and must be evaluated in individual bases.

Meat products had consistent DMD values during the gastric phase (average 41 %), except for the Meat Product 1 that a DMD of only 14·3 % (Table 1). This treat was made of whole pig ear that is primarily made of cartilage and collagen, which could explain its low gastric DMD. At the intestinal phase, however, this treat had a high DMD of 90·1 % and it did not differ from Meat Product 2 that was completely disintegrated with no residue observed. It is possible that the longer exposure to water and enzymatic solution during the intestinal phase may have optimised the action of digestive enzymes and improved the DMD of Meat Product 1. A study conducted in rats demonstrated that the total tract digestibility of collagen was near to 100 %^(^^4^^)^. All treats from the Meat Product category had a significantly greater DMD after 18 h of *in vitro* incubation (*P* < 0·05) when compared with the gastric phase. At the intestinal phase, Meat Products 3, 4 and 5 had a significantly lower DMD (average 74·3 %) when compared with Meat Product 2 (*P* < 0·05). This outcome was surprising because the latter was an extruded rawhide braid, whereas the former treats were chicken and duck jerkies.

The Rawhide category had a wide range of gastric DMD values, with Rawhide 1 being the least digestible (14·2 %), Rawhide 2 being the most digestible (73·1 %) and Rawhide 3 showing intermediate DMD (40·9 %; *P* < 0·05). At the intestinal phase, a similar pattern was observed. No statistical differences between Rawhides 2 and 3 were observed (DMD > 99 %), in contrast to Rawhide 1 that had a lower (*P* < 0·05) DMD (77·5 %). The use of slowly digestible treats, in this case reflected by a low gastric *in vitro* DMD, should be discouraged for dogs that tend to consume large pieces of food without much mastication prior to swallowing, as it could pose a risk for gastric blockage.

Overall, the Bone category was the least digestible among all treat categories tested in the present study (Fig. 1). On average, treats from Biscuit, Meat Product and Rawhide categories were less digestible at the gastric phase when compared with the intestinal phase (*P* < 0·05), whereas Chew and Dental treats were, on average, highly digestible and no significant differences between gastric and intestinal phases were observed (Fig. 1). In the present study, the DMD was determined using the intact (whole) treat; therefore, differences in *in vivo* DM digestibility could be observed due to the effect and extent of the animal chewing on the treat prior to its ingestion.

To our knowledge, very limited information on *in vitro* DMD and *in vivo* DM digestibility of treats is available. Only one previous study has examined the *in vitro* DMD and *in vivo* DM and nutrient digestibilities of expanded pork skin and rawhide treats^(^^5^^)^. In that study, a DMD of 54·7 and 7·6 % was observed for expanded pork skin chews and rawhide chews, respectively, after 6 h of incubation with HCl–pepsin solution. After an additional 18 h of incubation with pancreatin:phosphate buffer, the DMD of the expanded pork skin and rawhide increased to 99·0 and 70·1 %, respectively^(^^5^^)^. While a direct comparison between those results and those of the present study is difficult because the differences in ingredient composition and processing conditions are likely, a comparable average rawhide treat DMD after 18 h of *in vitro* incubation was observed between that study and the present one (88·1 and 91·9 %, respectively). In addition, ingestion of expanded pork skin treat resulted in greater DM and N digestibilities, as well as lower serum cholesterol and TAG concentrations, in adult dogs^(^^5^^)^.

In conclusion, understanding the DMD of commercially available treats is important to verify their safety for consumption and potential digestibility. The information gathered herein will assist in educating pet food formulators, veterinarians and pet owners about potential digestibility and safety of treats commonly purchased and fed to companion animals. In addition, the data emphasise the importance of providing adequate feeding guidelines for treats. Treat manufacturers must base their treat recommendations on purpose of use, predicted digestibility and treat size. The latter should mainly be considered when developing treats to accommodate a variety of body sizes, especially if a particular treat has a lower gastric DMD, which could increase the chances of gastric blockage or gastrointestinal distress. Additional work is necessary to bridge the lack of knowledge available on the safety and digestibility of commercially available treats. Once *in vitro* DMD data are available suggesting their safety, *in vivo* DM digestibility of commercially available treats should be evaluated as well as their potential impact in the overall nutritional when fed to dogs.

## Supplementary material

The supplementary material for this article can be found at http://www.journals.cambridge.org/jns
